# Liver Disease in Singapore

**DOI:** 10.5005/jp-journals-10018-1262

**Published:** 2018-05-01

**Authors:** Mark Muthiah, Chern H Chong, Seng G Lim

**Affiliations:** 1 Division of Gastroenterology and Hepatology, National University Health System, Singapore and Department of Medicine, Yong Loo Lin School of Medicine, National University of Singapore, Singapore; 2 Division of Gastroenterology and Hepatology, National University Health System, Singapore; Division of General Medicine, Woodlands Health Campus, Singapore

**Keywords:** Alcoholic liver disease, Burden of disease, Chronic hepatitis B, Chronic hepatitis C, Cirrhosis, Hepatocellular carcinoma, Liver cancer, Nonalcoholic fatty liver disease.

## Abstract

Liver disease is a significant health issue in Singapore. In the Singapore Burden of Disease Survey, liver cancer and liver cirrhosis contributed 3.2 and 0.9% of years of life lost (YLL) out of 182,753 YLL respectively. Liver cancer was ranked 8th and liver cirrhosis was ranked 20th in YLL. Liver cancer is the 5th most common cancer in males, and has an age-adjusted rate of 17.6 per 100,000 population. The underlying etiology of liver cirrhosis is chronic hepatitis B (CHB) in 63.3%, alcohol in 11.2%, cryptogenic in 9%, and chronic hepatitis C (CHC) in 6.9%. The overall seroprevalence rate of CHB is 3.6%, while CHC is approximately 0.1%. The trend in prevalence of liver cancer is gradually reducing as is CHB. However, less is known about alcoholic liver disease and fatty liver disease and there is some evidence that the latter is increasing. Singapore has a multilayered health care system designed to provide basic health care needs to the population. There are various schemes available that provide subsidized and assisted health care for treatment of hepatitis B and C as well as liver transplantation. Health policy with regard to a national action plan has not yet been developed and there is room for health care specialists, government and nongovernment agencies to work together to tackle liver disease in Singapore.

**How to cite this article:** Muthiah M, Chong CH, Lim SG. Liver Disease in Singapore. Euroasian J Hepato-Gastroenterol 2018;8(1):66-68.

## INTRODUCTION

A systematic review from the Global Burden of Disease Survey identified more than 1 million deaths due to liver disease globally.^1^ There were considerable variations of risk factors across various regions, with the main risk factors being hepatitis B and C infection, alcohol, schistosomiasis, and diabetes. The main causes of mortality from liver disease are liver cirrhosis and liver cancer. Hence, these two outcomes provide an overall profile of the status of liver disease in each country.

## EPIDEMIOLOGY

### Liver Cirrhosis in Singapore

In the Singapore Burden of Disease Survey,^2^ in the YLL, out of 182,753, liver cirrhosis contributed 0.9%. In hospitalized cases of cirrhosis, 27.6% causes listed were alcohol related and 68.5% were nonalcohol related. Based on expert opinion and methods in the Australian Burden of Disease Survey 2003, we attributed 5% of nonalcohol-related cirrhosis to other causes and the remainder to hepatitis (i.e., 63.5%). We assumed that the number of hepatitis B-related cirrhosis was about 300 times that for hepatitis C-related cirrhosis, based on estimated hepatitis B to hepatitis C carriers’ rates in the population. Hence, we estimated that 63.3% of cirrhosis cases were due to hepatitis B and 0.2% due to hepatitis C. As this was a cross-sectional study, no information on trends was reported. In a study of patients with liver cirrhosis from a hospital-based population in Singapore,^3^ the main etiologies of liver cirrhosis were CHB (63.3%), alcohol (11.2%), cryptogenic (9%), and CHC (6.9%). Although there are no current data on trends in liver cirrhosis, there is evidence that nonalcoholic fatty liver disease is likely to contribute a greater proportion of patients over time, particularly since with very effective hepatitis B vaccine program, the proportion of cases of CHB will fall over time.

### Liver Cancer in Singapore

This is the 5th most common cancer in males with an age-standardized rate of 16.8%.^4^ In the Singapore Burden of Disease Survey 2010,^2^ liver cancer contributed 5,886 patients to disability adjusted life years (DALYs) which was 1.5% of the total DALYs, and 5,783 YLL, being 3.2% of YLL. The etiology of hepatocellular carcinoma (HCC) is less clearly documented but in the largest series of HCC (n = 405) in 2015 that underwent surgical resection,^5^ 55.6% had CHB, 6.2% had CHC, 0.7% had both, and 31.9% had neither marker. This is consistent with the cause of cirrhosis previously shown. With regard to liver cancer trends, between 1973 and 2012, the incidence of liver cancer in Singapore declined steadily. The incidence in males dropped from 27.4 per 100,000 during 1973 to 1977 to 17.2 per 100,000 during 2008 to 2012, while the incidence in females decreased from 6.9 per 100,000 during 1973 to 1977 to 4.8 per 100,000 in the period 2008 to 2012. The reasons for this reduction are not clear but the report suggests a combination of factors particularly with regard to CHB—increasing effectiveness of hepatitis B virus (HBV) vaccination and better screening. Liver cancer in Singapore is ranked as the 4th most common cancer in men^6^ by the Singapore Cancer Registry and the 5th most common cancer in men between 2011 and 2015 by the Ministry of Health, Singapore, with an age-adjusted rate of 17.6 per 100,000.^7^ In the burden of disease survey for Singapore in 2010, liver cancer was ranked 8th (3.2%), and liver cirrhosis was ranked 20th (0.9%) as the cause of YLL.^2^

### Chronic Hepatitis B

In Singapore, the most recent community study reported an overall seroprevalence of 3.6% in adults aged 18 to 69 years in 2010.^8^ The ethnic distribution of CHB was highest in Chinese (4.2%), followed by Malays (2.2%) and Indians (0.6%). Estimates of the actual number of patients in care are unclear. In a study of CHB patients who were offered health screening in 2003, 67% of patients were found not to be on regular follow-up.^9^ In a follow-up study, the barriers to regular follow-up included lack of education, cost of follow-up, blood taking, and lack of time.^10,11^ Regular seroprevalence surveys for CHB show a steadily decreasing trend in seroprevalence.

As CHB is the most common cause of liver disease in Singapore, efforts to control and eradicate HBV are the highest priority. Singapore was one of the first countries in Asia to adopt universal HBV vaccination and this important development has led to a significant reduction in prevalence rates of CHB over time. However, patients with CHB cannot benefit from vaccination, hence efforts have been focused to improve their management.

Treatments approved for CHB in Singapore include all nucleos(t)ide analogs, lamivudine, adefovir, tenofovir, telbivudine, and entecavir. In addition, pegylated interferon alfa 2a is approved for CHB. These antiviral therapies are able to control CHB but not able to achieve functional cure, now defined as surface antigen of the hepatitis B virus loss. Consequently, we now have a robust research program directed toward functional cure. This is led by Professor Lim SG who was awarded a $25 million grant from the National Medical Research Council of Singapore for eradication of HBV.

### Chronic Hepatitis C

For CHC, a small sero-epidemiology study was performed in 1991 with first-generation antihepatitis C virus (HCV) enzyme-linked immunosorbent assay, and a seroprevalence of 1.7% was found in 463 “normal” individuals, 20% of hemodialysis patients, and 33% of patients with cirrhosis with no attributable cause.^12^ There has been no well-conducted community study of HCV prevalence, but estimates from the Ministry of Health approximate it to be 0.1%.^13^ A study found the HCV sero-prevalence rate of 0.059% among 161,658 blood donors between 2011 and 2014,^14^ and in hemodialysis patients, the rate was 2.2% among 1,575 patients. A more recent study of patients with liver cirrhosis found that 6.9% of all patients with liver cirrhosis at a tertiary liver center had HCV.^3^

### Nonalcoholic Fatty Liver Disease

There are no formal studies of prevalence of fatty liver disease in Singapore but at a public health forum, 40% in 227 subjects were diagnosed to have fatty liver.^15^ In a study of fatty liver in patients undergoing cholecystectomy, the prevalence increased over time in a 10-year period^16^ from 40 to 56.6%. In a large prospective cohort study, the Singapore Chinese Health Study, 63,257 patients were followed since 1993 to 1998 for a mean of 14 years of which 499 developed HCC. Diabetics had a hazard ratio of 2.14 (95% confidence interval 1.69-2.71) of developing HCC. Interestingly, this effect was independent of markers for viral hepatitis. In this study, a history of diabetes was present in 8.9% of the population at baseline. The Singapore Ministry of Health statistics inform us that 11.3% of Singaporeans have diabetes in 2010, which had increased from 8.2% in 2004. Since diabetes is a major risk factor for fatty liver and nonalcoholic steatohepatitis other than obesity, this provides some insight into an emerging and increasing serious health problem. The Ministry of Health also found that there is an increasing trend of obesity from 6.9% in 2004 to 10.8% in 2010 and 8.6% in 2013.

### Alcoholic Liver Disease

There are scanty data on alcoholic liver disease in Singapore but the Singapore Burden of Disease survey 2010 shows that there were 27.6% alcohol-related and 68.5% nonalcohol-related hospitalized cases of liver cirrhosis between 1991 and 2010.^2^ In a study on liver cirrhosis in a major public hospital in Singapore,^3^ out of 564 patients, 11.2% were due to alcoholic liver disease. Consequently, alcoholic liver disease can be considered a small but significant cause of liver cirrhosis.

## CURRENT HEALTH CARE POLICIES AND RESOURCES

Chronic hepatitis B is by far the most common problem and the mainstay of this is the National Hepatitis B Childhood Immunisation program implemented in 1987. With regard to those who are already identified as CHB patients, the Singapore hepatitis B guidelines were launched by the Ministry of Health in 2011,^17^ and provides guidance for the management of CHB in Singapore.

With regard to liver transplantation, there are four liver transplant centers in Singapore, two in the private sector and two in the public sector. From 2007 to 2016, a total of 261 liver transplantations were performed.^18^ About 105 cases were living donor liver transplants, while 156 cases were deceased donor liver transplants.

## CONCLUSION

The key to developing a comprehensive strategy to tackle liver disease is to identify the extent of the problem and the burden of disease. While liver disease as a whole does not appear to be a huge problem in Singapore, the finding that liver cancer is the 5th most common cancer in men, and liver cirrhosis contributed 0.9% of YLL in the 2010 Singapore Burden of Disease Survey, suggests that it is still a significant problem.
